# Hyperuricemia; a new look at an old problem

**Published:** 2012-07-01

**Authors:** Saeed Mardani, Hamid Nasri, Mahmoud Rafieian-Kopaei, Seyed Seifollah Beladi Mousavi

**Affiliations:** ^1^Department of Nephrology, Shahrekord University of Medical Sciences, Shahrekord, Iran; ^2^Department of Nephrology, Division of Nephropathology, Isfahan University of Medical Sciences, Isfahan, Iran; ^3^Medical Plants Research Center, Shahrekord University of Medical Sciences, Shahrekord, Iran; ^4^Department of Internal Medicine, Ahvaz Jundishapur University of Medical Sciences, Ahvaz, Iran

**Keywords:** Hyperuricemia, Diabetic nephropathy, IgA nephropathy

**Implication for health policy/practice/research/medical education:**

Studies have shown that elevated uric acid levels have a deleterious effect, directing to endothelial dysfunction, inflammation, and vascular disease. Elevated uric acid initiates endothelial dysfunction by inhibiting nitric oxide synthetase, activating the renin-angiotensin system and causing pro-inflammation with leads to endothelial dysfunction, vascular smooth muscle cell dysfunction, and finally contributing to atherosclerosis and vascular dysfunction and aggravation of diabetic kidney disease and IgA nephropathy and other nephropathies. While various clinical investigations have shown that, lowering uric acid with allopurinol improved endothelial dysfunction in both hyperuricemic subjects and hypertensive type 2 diabetic patients with normal uric acid levels. We therefore suggest, further clinical studies, to better find the allopurinol kidney protective properties in diabetes, IgA nephropathy or other nephropathies.

Recently much attention had been directed toward the significance of hyperuricemia in general population and aggravation of nephropathy. Various investigations have shown that elevated uric acid levels might have a deleterious effect, directing to endothelial dysfunction, inflammation, and vascular disease (1,2). The proposed mechanisms is that elevated uric acid initiates endothelial dysfunction by inhibiting nitric oxide synthetase, activating the renin-angiotensin system and causing pro-inflammation with leads to endothelial dysfunction, vascular smooth muscle cell dysfunction, and finally contributing to atherosclerosis and vascular dysfunction (1–3). About 70% of uric acid is removed by the kidneys, hyperuricemia, therefore happens when renal function deteriorates. It is not fully understood that whether the hyperuricemia, observed in renal failure, plays a role in the progression of kidney failure (1–4). Recent clinical findings demonstrated that, the serum uric acid is narrowly linked to hypertension (HTN) in hyperuricemic subjects and also with the beginning of HTN (2–4). In fact, uric acid, as the final oxidation product of purine catabolism, has been associated with various clinical conditions such as diabetes and atherosclerotic disease too. Recent findings suggest that uric acid is a relevant and independent risk factor for kidney disease, particularly in patients with HTN (1–5). It was observed that hyperuricemia, when induced by an uricase inhibitor, initiates HTN and impairs nitric oxide generation in the macula densa of the glomerulus, while both HTN and kidney damage are reduced by inducing of nitric oxide (1–5). The mechanism by which uric acid may cause organ injury is not fully defended, nevertheless there is increasing evidence that dysfunction of endothelial cells as mentioned above is a mechanism whereby this substance may affect kidney function and structure. HTN is consistently linked to endothelial dysfunction and hyperuricemia is a strong predictor of HTN and blood pressure progression (1,3–5). Though, irrespective of kidney involvement, elevated serum uric acid is related with renal development of hypertension. It was also shown that in the adolescents with newly diagnosed hypertension, treatment with allopurinol resulted in lowering of blood pressure. On the other hand, it is well recognized that hyperuricemia is an independent risk factor for IgA nephropathy too, and appropriate treatment by allopurinol is a reasonable modality in this group (6). In fact, this drug can routinely be added to the treatment of IgA nephropathy or diabetics patients (4–6). Though various clinical investigations have shown that, lowering uric acid with allopurinol improved endothelial dysfunction in both hyperuricemic subjects and hypertensive type 2 diabetic patients with normal uric acid levels (3–8).We therefore suggest, further clinical studies, to better find the allopurinol kidney protective properties in diabetes, IgA nephropathy or other nephropathies.

## Authors’ contributions

All authors contributed to the paper equally.

## Conflict of interests

The authors declared no competing interests.

## Ethical considerations

Ethical issues (including plagiarism, misconduct, data fabrication, falsification, double publication or submission, redundancy) have been completely observed by the authors.

## Funding/Support

None declared.
